# The MindMoves Trial: Effect of Lifestyle Physical Activity and Cognitive Training Interventions on Cognition in Older Women with Cardiovascular Disease

**DOI:** 10.1002/alz.087735

**Published:** 2025-01-09

**Authors:** Shannon Halloway, Zoe Arvanitakis, Michael E Schoeny, Annabelle Santos Volgman, Susan J Pressler, Sachin Vispute, JoEllen Wilbur, Lisa L. Barnes

**Affiliations:** ^1^ University of Illinois Chicago College of Nursing, Chicago, IL USA; ^2^ Rush Medical College, Chicago, IL USA; ^3^ Rush University Medical Center, Chicago, IL USA; ^4^ Rush University College of Nursing, Chicago, IL USA; ^5^ Indiana University School of Nursing, Indianapolis, IN USA; ^6^ Rush Alzheimer’s Disease Center, Chicago, IL USA

## Abstract

**Background:**

Cardiovascular disease (CVD) increases the risk of cognitive impairment (CI), particularly in women. Physical activity and cognitive training can improve cognition, with potential interactive effects. The purpose of this study was to evaluate the efficacy of *MindMoves*, a 24‐week multidomain physical activity and cognitive training intervention, on cognition in older women with CVD.

**Method:**

This randomized controlled trial (NCT04556305) with a 2×2 factorial design tested the independent and combined efficacies of *Mind* (tablet‐based cognitive training) and *Move* (lifestyle physical activity with goal‐setting and group meetings) on change in cognition (episodic memory, semantic memory, working memory, and executive function). Women (*n* = 253) aged 65 years and older with CVD and without CI at baseline were randomized to (1) *Mind*, (2) *Move*, (3) *MindMoves*, or (4) usual care. Intervention orientations were in‐person, and all other intervention activities were home‐based. From September 2020–December 2023, participants completed telephone neuropsychological tests and questionnaires at baseline, 24, 48, and 72 weeks.

**Result:**

Participants were, on average, 72.4 years old at baseline (*range* = 65‐90), primarily of non‐Hispanic White (54%) and non‐Hispanic Black (38%) racial backgrounds, and 60% graduated college. A total of 86% (*n* = 218) of participants completed the 72‐week data collection. Attrition was not a function of intervention condition, sociodemographics, or baseline cognition (all *p*’s > .05). On average, all participants showed improvement in episodic memory and executive function. In multilevel mixed models adjusted for age, education, and racial and ethnic background, the main effects of *Mind* and *Move*, and the interactive effects (Mind*Move interaction), on linear change over 72 weeks were not statistically significant for any of the four cognition outcomes. In additional models that tested the intervention effects averaged across all follow‐up timepoints, none of the intervention conditions were associated with cognition.

**Conclusion:**

Although participants improved in two domains of cognition, there were no differential effects of the intervention conditions (cognitive training and/or physical activity) in this cohort of older women with CVD. Future analyses will examine intervention effects on serum biomarkers that may provide additional insights of efficacy.

